# Vertebral Artery-Posterior Inferior Cerebellar Artery (PICA) Aneurysm Treated With the PICA-to-PICA Bypass: A Case Report

**DOI:** 10.7759/cureus.76863

**Published:** 2025-01-03

**Authors:** Yusuke Sakamoto, Osamu Suzuki, Toshiki Fukuoka, Takayuki Awaya, Ryuta Saito

**Affiliations:** 1 Neurosurgery, Japanese Red Cross Aichi Medical Center Nagoya Daini Hospital, Nagoya, JPN; 2 Neurosurgery, Nagoya Ekisaikai Hospital, Nagoya, JPN; 3 Neurosurgery, Nagoya University Hospital, Nagoya, JPN

**Keywords:** anastomosis, aneurysm, bypass, clipping, posterior inferior cerebellar artery, vertebral artery

## Abstract

Treating vertebral artery-posterior inferior cerebellar artery (VA-PICA) aneurysms poses challenges because of their complex anatomy. Although endovascular treatment is commonly preferred, direct surgery offers better recovery prospects than endovascular surgery aided by bypass techniques. We present a VA-PICA aneurysm case treated with VA internal trapping, using a PICA-to-PICA bypass without direct clipping. A 64-year-old man presented with complaints of severe headache and was diagnosed with a right VA-PICA wide neck aneurysm. The PICA originated from the neck of the aneurysm. We consulted an endovascular surgeon and opted for direct clipping via the transcondylar fossa approach. During surgery, we secured and followed the right VA, identified the PICA origin, and observed the proximal neck of the aneurysm. However, securing the distal VA was challenging because the dissector was inaccessible. Instead of direct clipping, a PICA-to-PICA bypass was selected following internal trapping. After anastomosis, the proximal PICA was occluded, whereas the small perforating artery was preserved. The next day, internal trapping of the right VA was performed. The patient’s postoperative course was uneventful, except for mild dysphagia. Head magnetic resonance imaging revealed a small infarction on the right medulla. Three-dimensional computed tomography angiography revealed perfect PICA-to-PICA bypass patency. The patient was subsequently transferred to a rehabilitation hospital. The patient had no neurological symptoms at the six-month follow-up (Modified Rankin Score = 0).

The preoperative feasibility of direct clipping of complex PICA aneurysms while preserving PICA is unpredictable. Various bypass methods, including the PICA-to-PICA bypass, are potential treatment options for complicated VA-PICA aneurysms.

## Introduction

Treating vertebral artery (VA)-posterior inferior cerebellar artery (PICA) aneurysms remains challenging owing to their depth, important surrounding elements, including the lower cranial nerve and perforating arteries to the brainstem, complexed structure of aneurysm depending on the anatomical variation of PICA, and obstructive bony structure [1-3]. Endovascular treatment is the preferred option in several institutions owing to its minimally invasive nature and accessibility to the aneurysm; however, complex cases, broad neck type, involving PICA origin, may necessitate direct surgery to preserve PICA or availability of vascular reconstruction [4,5]. Although direct clipping surgery is the ideal treatment for saccular aneurysms, its feasibility is uncertain preoperatively. The occipital artery (OA)-PICA bypass offers multiple treatment options for an aneurysm in this lesion when PICA reconstruction is required. However, preparation of the occipital artery for every case is highly invasive and time-consuming [6,7]. Depending on the intraoperative findings and surgical environment, direct clipping may be omitted. In such scenarios, intracranial-to-intracranial bypass surgery allows for flexible adaptation to trapping or internal trapping surgery, despite potential pitfalls associated with such a bypass [5,8]. Herein, we present a case of VA-PICA aneurysm treated with VA internal trapping aided by a PICA-to-PICA bypass following the decision to avoid direct clipping.

This article was previously presented as an oral presentation at the 53rd Annual Meeting of the Japanese Society for Surgery of Cerebral Stroke, March 7-9, 2024, in Yokohama, Japan.

## Case presentation

**Figure 1:**
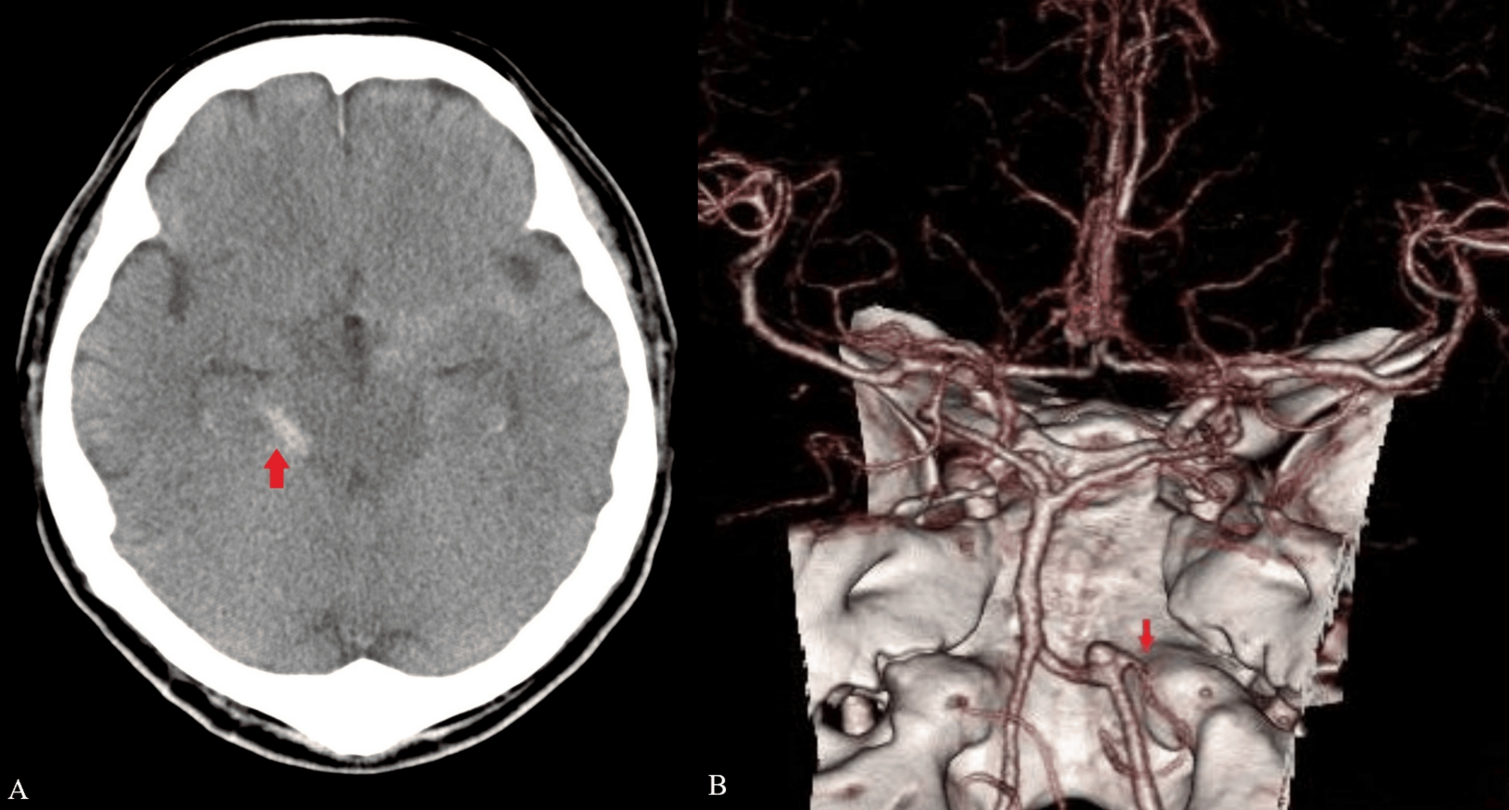
Preoperative head computed tomography and three-dimensional computed tomography angiography. (A) Computed tomography of the head showed Fisher 2 subarachnoid hemorrhage (arrow). (B) Three-dimensional computed tomography angiography demonstrating right vertebral artery-PICA aneurysm. Note the PICA originated from the neck of the aneurysm (arrow). PICA: posterior inferior cerebellar artery

A 64-year-old man was delivered to our hospital with the chief complaint of sudden onset of severe headache. The patient’s level of consciousness on admission was E3V5M6 according to the Glasgow Coma Scale. Computed tomography (CT) of the head demonstrated Fisher 2 subarachnoid hemorrhage (Figure 1A). Three-dimensional computed tomography angiography (CTA) revealed a right VA-PICA aneurysm (Figure 1B).

The right VA deviated medially and the PICA originated from the aneurysm neck, which was wide. Under this situation, potential treatments were as follows: flow diverter in the right vertebral artery, flow diverter from the vertebral artery into PICA, balloon-assisted coiling, balloon test occlusion with vertebral artery sacrifice, primary clipping, proximal PICA clipping with OA-to-PICA or PICA-to-PICA, and PICA excision reimplantation. The treatment strategy was discussed with the endovascular surgeon; however, PICA thrombosis raised concerns about endovascular treatment consequences. Also, stent graft including flow diverter for the subarachnoid hemorrhage acute phase is off-label under the Japanese health insurance system. Parent artery occlusion/clipping with or without bypass is one of the treatment options, but the inevitable blind pouch might bring perforating artery thrombosis. Ultimately, we opted to perform clipping the following day.

**Figure 2:**
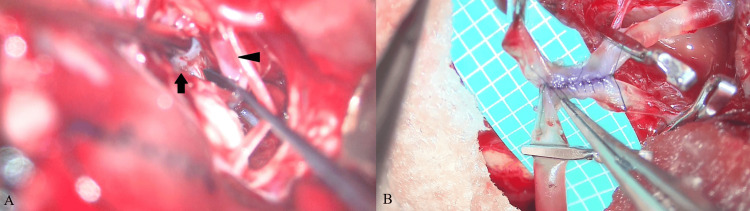
Overcoming clipping challenges and ensuring recovery with a PICA-to-PICA bypass. (A) Proximal vertebral artery (arrow) and PICA (arrowhead) are shown. The distal vertebral artery is not visible. (B) PICA-to-PICA anastomosis. PICA: posterior inferior cerebellar artery

**Figure 3:**
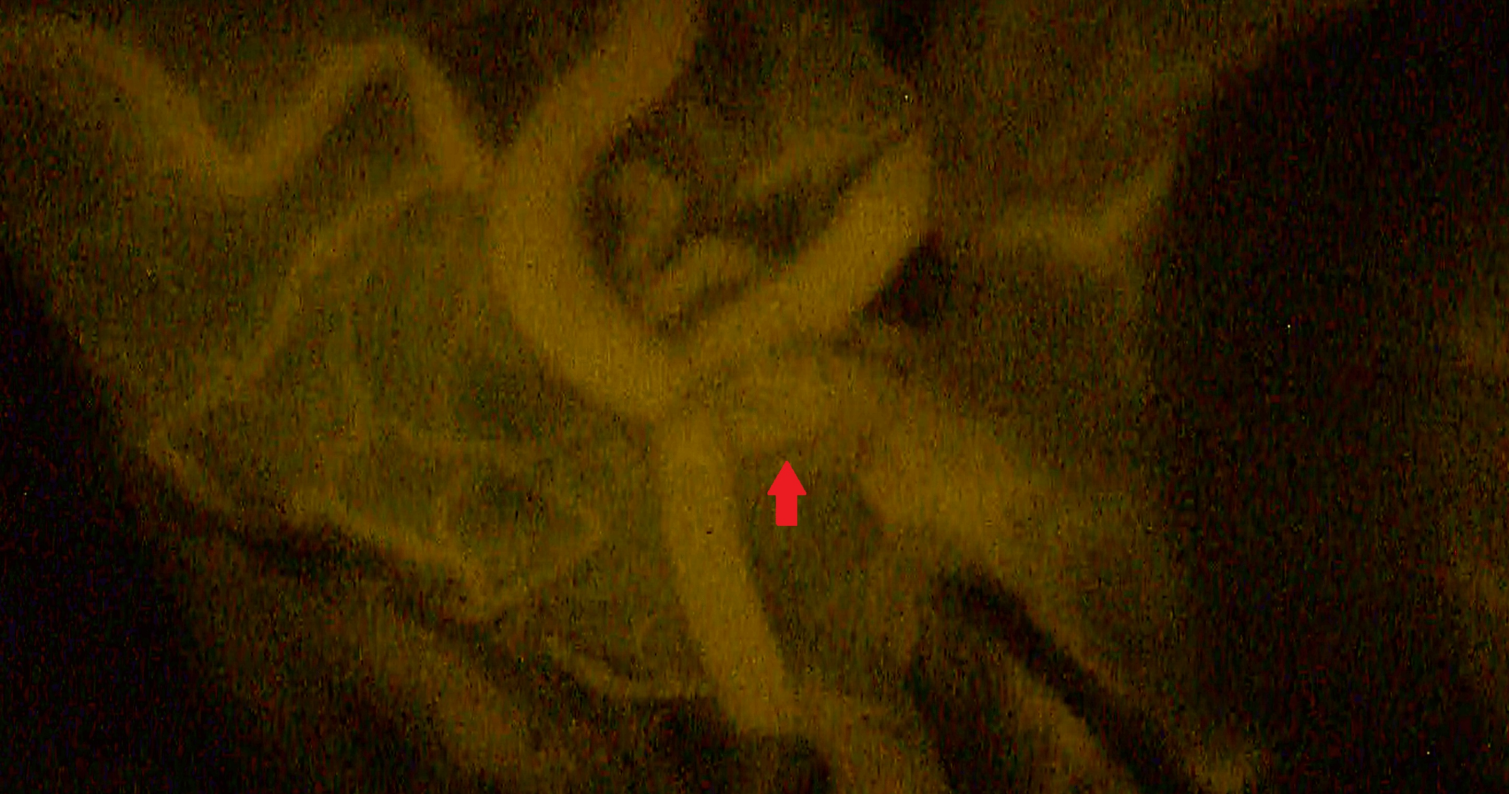
Intraoperative indocyanine green test. The test demonstrates good patency of the PICA-to-PICA bypass (arrow). PICA: posterior inferior cerebellar artery

A right hockey stick skin incision and craniotomy were performed under general anesthesia and in the prone position. The condylar fossa was drilled until the hypoglossal canal opened. The posterior part of the jugular tubercle was visualized under a microscope. After opening the dura mater, the cerebellomedullary fissure was widely opened, and the cerebellum was gently retracted rostrally. We secured and followed the right VA and identified the PICA origin; however, the distal VA could not be observed (Figure 2A). We continued dissection but finally found it extremely challenging because the jugular tuberculum was obstructive and the aneurysm was located at such a depth that our dissector could not reach. The right VA had a steep medial turn after the PICA origin, which made it difficult to secure the right distal VA. In this scenario, premature rupture would lead to uncontrolled bleeding. We considered direct clipping as an alternative. Occipital artery (OA)-PICA bypass was not applicable, as the OA had already coagulated. The bilateral PICAs were positioned parallel, and the diameters of these arteries were well-matched. We decided to perform a PICA-to-PICA bypass. A running suture was performed on the back wall, and intermittent sutures were performed on the front wall with a 10-0 nylon (Figure 2B). The total clamp time for the PICA was 36 min. After declamping, an indocyanine green test demonstrated good PICA-to-PICA bypass patency (Figure 3).

**Figure 4:**
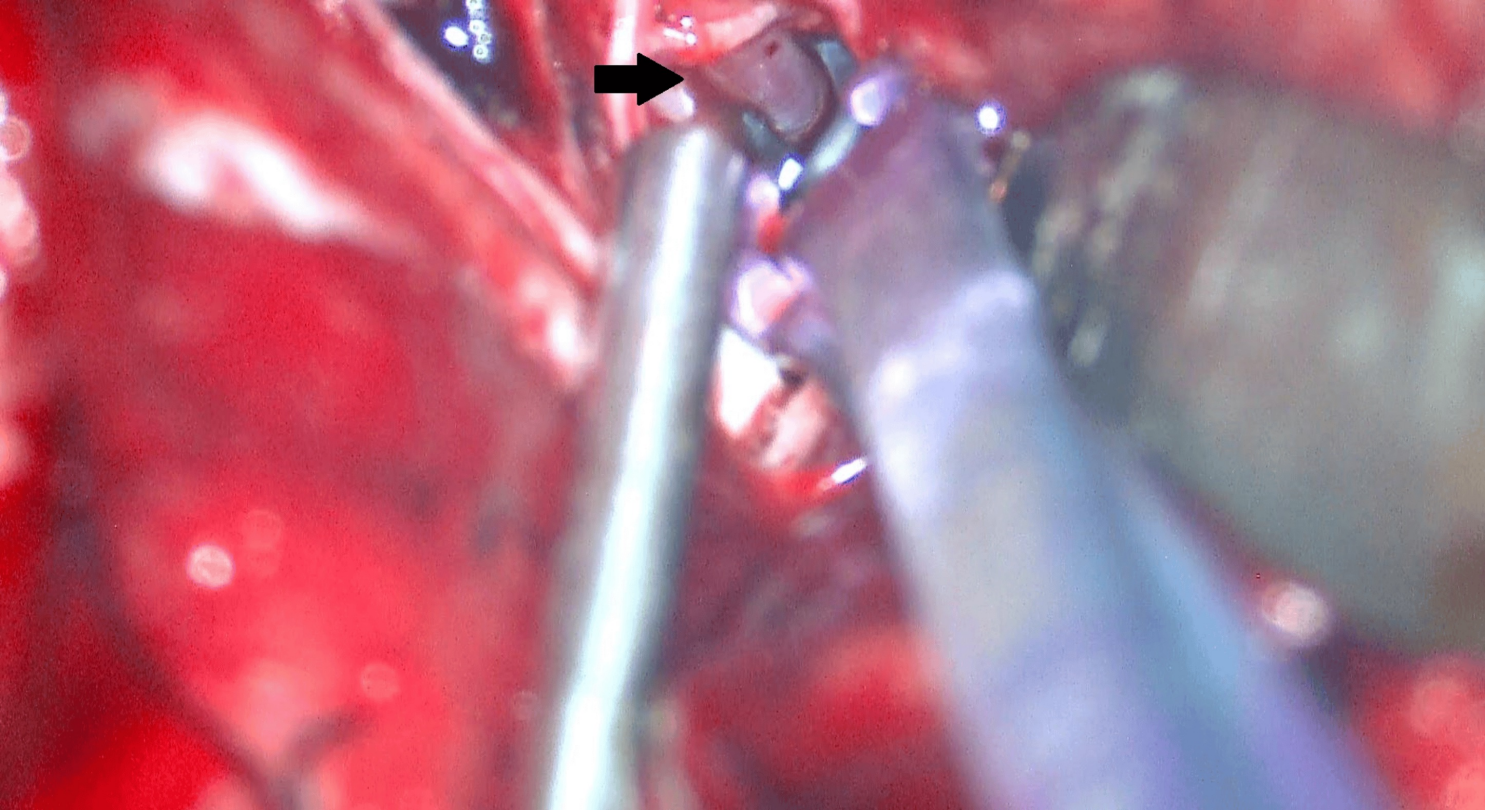
Occlusion of the PICA distal to the aneurysm The PICA distal to the aneurysm was occluded using a titanium clip (arrow). PICA: posterior inferior cerebellar artery

**Figure 5:**
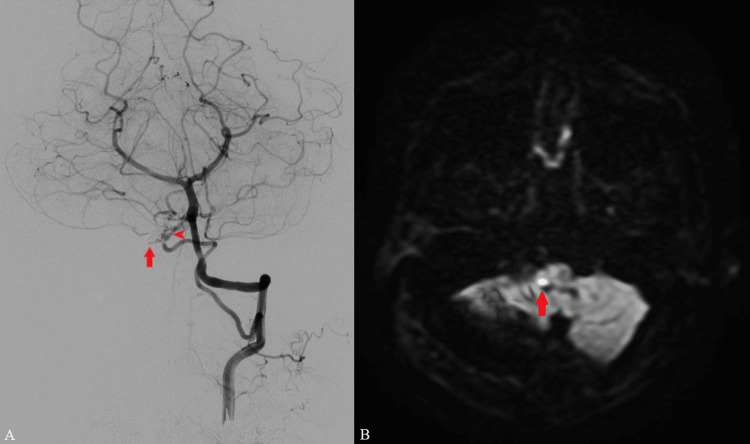
Postoperative left vertebral angiography and diffusion-weighted head magnetic resonance imaging. (A) Postoperative left vertebral angiography. Sacrifice of the vertebral artery-PICA aneurysm together with the right vertebral artery was performed (arrow). Right PICA was seen through the PICA-to-PICA anastomosis (arrowhead). (B) Diffusion-weighted head magnetic resonance imaging revealed a small infarction in the right medulla (arrow). PICA: posterior inferior cerebellar artery

The PICA distal to the aneurysm was occluded using a titanium clip to avoid bypass thrombosis owing to antegrade blood flow from the right PICA (Figure 4). The total operative time was 467 min. The following day, endovascular surgeons performed internal trapping of the right VA under general anesthesia. A 6-Fr catheter was guided and placed in the right VA via the right femoral artery, and a 4-Fr diagnostic catheter was guided to the left VA via the left femoral artery. Initially, we tried to perform saccular packing but encountered difficulty. Therefore, we decided to embolize the right VA including the aneurysm (Figure 5A). The patient was extubated on day three and exhibited dysphagia and hoarseness. Diffusion-weighted head magnetic resonance imaging revealed a small infarction in the right medulla that seemed to be responsible for these symptoms (Figure 5B).

This infarction might have been caused by perforating artery thrombosis due to sacrifice of vertebral artery or blind allay of the proximal PICA. The patient’s symptoms gradually improved; however, mild dysphagia persisted. The patient was transferred to a rehabilitation hospital, and they recovered well and had no neurological symptoms at the six-month follow-up at our outpatient clinic (Modified Rankin Score = 0).

## Discussion

VA-PICA aneurysms are primarily treated using endovascular methods because they are less invasive and more accessible [9,10]. However, some complex aneurysms, broad neck type, involving PICA origin, and large thrombosed type, cannot be treated with simple embolization [11]. Although stent-assisted coil embolization is a potential treatment option, ischemic complications can occur [12]. Moreover, this treatment option for the subarachnoid hemorrhage acute phase is off-label under the Japanese health insurance system.

In the present case, we selected direct surgery after consulting with the endovascular surgeon and initially attempted to perform a clipping operation. Unfortunately, intraoperative neck clipping was challenging to complete because of the obstructive jugular tuberculum, deep aneurysm location, and the right VA steep medial turn after the PICA origin, which made it difficult to secure the right distal VA. We decided to perform a PICA-to-PICA bypass followed by internal trapping, as the OA had already coagulated. The greatest advantage of direct surgery is the possibility of vascular reconstruction, including the OA-PICA bypass, PICA-to-PICA bypass, and PICA transposition [13,14]. Presuming preoperative feasibility, intraoperative execution of direct clipping while preserving the PICA can be extremely challenging. Despite these circumstances, we performed clipping with PICA sacrifice and bypass assistance. If neither clipping nor trapping were difficult to perform intraoperatively, we could pivot to a combined treatment strategy with bypass assistance. Therefore, bypass can increase the chances of recovery [5]. Endovascular embolization is undoubtedly more beneficial for VA-PICA aneurysms than direct surgery as a less invasive procedure and has better accessibility. However, in complicated cases, endovascular treatment may not provide the best chances of recovery. Parent artery occlusion without vascular reconstruction may bring ischemic complications to some extent [15]. While endovascular treatment has become more prevalent, direct surgery for posterior inferior cerebellar artery aneurysms should be reconsidered as the primary treatment option due to its ease of flexible recovery during complications, the availability of vascular reconstruction, and the less ischemic complication [16]. The OA-PICA bypass offers greater versatility in vascular reconstruction for this lesion than the PICA-to-PICA bypass [17]. The feasibility of the PICA-to-PICA bypass depends on anatomical considerations [4,8]. In the present case, preparing for an OA graft before craniotomy might have been advantageous. However, the feasibility of direct clipping before craniotomy is uncertain. In addition, harvesting the OA is troublesome, time-consuming, and invasive because of its tortuous course [18]. Thus, the PICA-to-PICA bypass was crucial for maximizing the chance of recovery in this case, despite inherent risks such as contralateral ischemic complications. The PICA-to-PICA bypass is a potential treatment option, particularly for complicated VA-PICA aneurysms.

Direct surgery utilizing vascular reconstruction including PICA-to-PICA bypass should be reconsidered as a prominent treatment option for posterior inferior cerebellar artery aneurysms. However, since the present report is based on a single case, further accumulation of cases is needed.

## Conclusions

The preoperative feasibility of direct clipping of complex PICA aneurysms, while preserving the PICA, is unpredictable. If neither clipping nor trapping were difficult to perform intraoperatively, the treatment strategy could be changed to a combined treatment with bypass assistance. Compared with endovascular treatment, direct surgery is more flexible to pivot the treatment strategy intraoperatively because of the availability of vascular reconstruction. Various bypass methods, including the PICA-to-PICA bypass, are potential recovery options, particularly for complicated VA-PICA aneurysm.
